# The Impact of Visual Impairment on Quality of Life

**Published:** 2016

**Authors:** Masoud KHORRAMI-NEJAD, Amin SARABANDI, Mohammad-Reza AKBARI, Farshad ASKARIZADEH

**Affiliations:** 1 Eye Research Center, Farabi Eye Hospital, Tehran University of Medical Science, Tehran, Iran; 2 Department of Optometry, School of Paramedical Sciences, Mashhad University of Medical Sciences, Mashhad, Iran

**Keywords:** Visual Impairment, Quality of Life, Zahedan, Visual Field (VF), Visual Acuity (VA), Stereopsis

## Abstract

Our goal was to identify and describe factors relating to quality of life (QOL) in subjects with low vision and blindness in Iran's Sistan and Baluchestan Province. This cross-sectional study was carried out in randomly selected subjects with vision disability who were covered by the Zahedan Welfare Organization in Zahedan, Iran. The following factors related to visual impairment were evaluated: visual field (VF), visual acuity (VA), and stereopsis. Data were collected using a demographic questionnaire and the Influence of Vision Impairment (IVI) questionnaire. One-hundred and twenty-one patients were enrolled for participation in the study. T-test analyses indicated that the mean QOL score for women was significantly lower than that for men (P < 0.001).

Mann-Whitney U tests indicated that mean social (P = 0.003) and leisure (P = 0.009) QOL scores were significantly lower in participants without stereopsis. In addition, participants with tunnel vision scored lower on the mobility and self-care categories (P < 0.001) than others. The results of this study indicate that providing education, providing employment, improving, and expanding social programs for the blind and individuals with low vision people, especially women, are necessary.

## INTRODUCTION

Blindness and visual defects lead to a variety of public health, social, and economic problems, especially in developing countries (1). The World Health Organization has declared that blindness and visual impairment affect 37 million and 124 million individuals worldwide, respectively (2). Over 90% of individuals with blindness and low vision live in developing countries (3). In the past decade, evaluations of health and eye care have increasingly focused on health-related quality of life (QOL) as a criterion for treatment (4). Recent studies have shown that visual disability affects a person’s QOL by limiting social interactions and independence (5, 6). Thus, evaluation of the influence of visual impairment on daily activities, emotional state, social participation, and mobility is very valuable. Research in this will facilitate better provision of services for individuals with blindness and impaired vision. The mobility domain of QOL is reduced in patients with low vision or blindness when compared to normal individuals (7). In fact, there is a monotonic relationship between changes in visual function and those in QOL (8). Social and economic conditions, personal characteristics, and the values and norms of indigenous and local populations are all factors affecting the impact of disease and health problems on a person’s daily activities and his or her QOL (9). Determining the influence of various factors associated with impaired vision on the QOL of patients with low vision or blindness in different countries and different cultures is thus necessary and very important. 

The province of Sistan and Baluchestan in southwestern Iran borders Afghanistan and Pakistan (1). Zahedan is the capital city of the province. Although disability is often reported as a characteristic of deprived individuals, and the detrimental influence of visual disability on QOL is well documented (10, 11), no studies have determined the impact of vision impairment in this area. The results of this survey can be used to organize rehabilitation programs to alleviate factors affecting QOL in patients with blindness and visually impairment. The main objective of this study was to determine the influence of visual function impairments and demographic factors on QOL in individuals with blindness and low vision living in Zahedan.

## MATERIALS AND METHODS

This cross-sectional study was conducted in 2015. All individuals with blindness and low vision covered by the Welfare Organization in Zahedan, Iran, were assessed. People aged 7 years or older with no other disability were included in the study. This study was approved by the Review Board at Tehran University of Medical Sciences and adhered to the principles of the Declaration of Helsinki. All study subjects signed an informed consent statement after a verbal account was provided to them regarding the aims and the methods of this study. The first step in this survey was to obtain the necessary authorization from the Zahedan Welfare Organization. Following that, eligible individuals were identified. The researcher then explained the research goals to those with vision disabilities and their families and asked for cooperation with the study. Individuals willing to cooperate were then sent to the Al-Zahra Eye Centre, Zahedan, Iran for examination. Factors related to visual impairment, such as visual acuity (VA), visual field (VF), and stereopsis were evaluated and measurements were obtained by an optometrist. Monocular assessment of distance VA was performed using a Logarithm of the Minimum Angle of Resolution (LogMAR) chart at a distance of 6 meters. VA was thus reported as corrected distance visual acuity. VA was categorized as follows: (in LogMAR value): no light perception, light perception, > 1.8, 1.8 to 1.40, 1.40 to 1, and 1 to 0.5 (4, 12). VF was assessed using the Goldmann perimeter (binocular) with the III/4e target at standard background luminance. Participants were placed in the blindness category if they had a VA of 1 or worse in the better eye or a VF diameter of 20 degrees or less in the better eye. Participants were placed in the low vision category if they had a VA between 1 to 0.5 in the better eye and VF diameter of more than 20 degrees in the better eye. This was in accordance with standard diagnostic criteria (4, 12).

A random dot stereo butterfly was used to measure stereoscopic vision. Participants were organized into those without stereopsis (Randot butterfly could not be identified, > 2,000-second arc), and those with stereoscopic vision (Randot butterfly identified, ≤ 2000-second arc) (13). After visual examination, the researcher asked each participant questions in a clear manner and then completed the demographic questionnaire. In addition, a questionnaire was administered to each blind individual regarding his or her QOL. Demographic variables included age, sex, education level, marital status, and employment status. Each participant’s QOL was assessed using the Impact of Vision Impairment questionnaire. This questionnaire was prepared by Frost et al. at the Royal Victorian Eye and Ear Hospital in 2000. Tavakol et al. translated the questionnaire into Farsi in 2007. Validity and reliability were assessed using the content validity and test-retest methods, respectively (14). The questionnaire had 46 questions regarding the following aspects of life: self-care (9 questions), leisure (8 questions), emotional health (13 questions), social life (8 questions), and mobility (8 questions). Response options for the classification questions were as follows: never, seldom, sometimes, most of the time, and all the time. Levels of evaluation ranged from 0 to 4.. In this manner, responses to the QOL questions were divided into 4 groups: undesirable, relatively desirable, desirable, and completely desirable (14). Finally, data analysis was performed using SPSS version 22. Central tendency and dispersion indices were used for descriptive statistics in the data analysis. T-tests, analyses of variance (ANOVAs), and correlation tests were used for parametric tests, and the Mann-Whitney U test was used for unpaired comparisons in non-parametric tests. All p-values less than 0.05 were considered significant.

## RESULTS

Two-hundred individuals with blindness or low vision were covered by the Zahedan Welfare Organization at the time of the study. One-hundred and twenty-one individuals were eligible for enrolment in this study. Of these individuals, 68 (56.2%) were men, and 53 (43.8%) were women. The average age of the men was 26.32 (± 12.98) and that for women was 21.04 (± 7.68). Ninety-six participants (79.3%) were blind and 25 participants (20.7%) had low vision. Other information is shown in Tables 1 and 2.

**Table 1:** Frequencies and mean (**± **SD) QOL scores in study subjects according to demographic factors

	**No. (%)**	**QOL scores, Mean ± SD**
**Marital**		
Married	24 (19.8)	110.75 ± 21.90
Single	97 (80.2)	90.29 ± 25.70
**Education**		
Diploma or lower than diploma	97 (80.2)	89.10 ± 24.66
Associate Degree	9 (7.4)	101.78 ± 19.62
Bachelor’s Degree or higher	15 (12.4)	123.80 ± 18.62
**Employment status**		
Employed	21 (17.4)	112.90 ± 19.76
Unemployed	100 (82.6)	90.45 ± 25.80

SD = standard deviation, QOL = quality of life, No. (%) = number (percent)

**Table 2:** Frequencies and mean (**± **SD) QOL scores in study subjects according to visual factors

	**No. (%)**	**QOL scores, Mean ± SD**
**Disability**		
Blind	96 (79.3)	94.70 ± 25.29
Low vision	25 (20.7)	93.00 ± 30.93
**VA (in LogMAR value) **		
N.L.P	13 (10.7)	116.62 ± 22.88
L.P	17 (14.0)	88.06 ± 22.36
> 1.8	22 (18.2)	86.14 ± 23.08
1.8 to 1.4	44 (36.4)	95.07 ± 24.92
1.4 to 1	25 (20.7)	93.00 ± 30.03
**Stereopsis**		
≤ 2,000 seconds of arc	9 (7.4)	112.11 ± 26.30
> 2,000 seconds of arc	112 (92.6)	92.92 ± 25.80
**VF**		
More than 20 degrees	46 (38.0)	96.93 ± 20.43
20 degrees or less	75 (62.0)	92.76 ± 29.23

SD = standard deviation, QOL = quality of life, No. (%) = number (percent), VA = visual acuity, VF = visual field, N.L.P. = no light perception, L.P. = light perception

The mean QOL score for women was significantly lower than the score for men (P < 0.001). However, no statistically significant differences were noted in QOL between subjects with blindness and those with low vision (P = 0.774). There were statistically significant differences between mean QOL scores of married vs. single individuals (P < 0.001). ANOVA indicated significant differences between individuals with different educational levels in mean QOL scores (P < 0.001) (Tables 3 and 4).

Our results indicate that most individuals (52.1%) had a relatively desirable QOL. The majority of participants (54.5%) had relatively desirable mobility QOL scores. At the same time, the majority of participants (63.6%) also had undesirable leisure QOL scores. Finally, the majority of participants (64.5%) had completely desirable self-care QOL scores (Table 5).

**Table 3:** Comparison of QOL scores according to demographic variables

	**QOL scores, Mean ± SD**	**df**	**P-value**	**95% CI of the difference**	
				**Lower**	**Upper**
**Sex**		119	< 0.001	17.5	34.15
Male	105.66 ± 22.65				
Female	79.83 ± 23.30				
**Marital status**		119	< 0.001	13.75	9.17
Married	110.75 ± 21.90				
Single	90.29 ± 25.70				
**Employment status**		119	< 0.001	10.62	34.29
Employed	112.90 ± 19.76				
Unemployed	90.45 ± 25.80				

SD = standard deviation, QOL = quality of life, CI = confidence interval, df= degrees of freedom

**Table 4:** Comparison of QOL scores according to education level

**Educational level**	**No. (%)**	**QOL scores, Mean ± SD**	** df**	**Sig.**
**Diploma and low literacy**	97 (80.2)	89.10 ± 24.66	2	< 0.001
**Associate Degree**	9 (7.4)	101.78 ± 19.62	118	
**Bachelor's Degree or higher**	15 (12.4)	123.80 ± 18.62	120	

SD = standard deviation, QOL = quality of life, CI = confidence interval, N (%) = number (percent), Sig. = significance, df= degrees of freedom

**Table 5:** Frequencies and mean (**± **SD) QOL scores in different domains

**Domain**	**Undesirable, No. (%)**	**Relatively desirable, No. (%)**	**Desirable, No. (%)**	**Completely desirable No. (%)**	**Mean ± SD**
**Self-care**	1 (0.8)	1 (0.8)	41 (33.9)	78 (64.5)	28.51 ± 4.01
**Leisure**	77 (63.6)	36 (29.8)	8 (6.6)	0 (0.0)	7.80 ± 5.32
**Mobility**	14 (11.6)	66 (54.5)	40 (33.1)	1 (0.8)	14.17 ± 5.70
**Social**	50 (41.3)	49 (40.5)	22 (18.2)	0 (0.0)	10.23 ± 5.10
**Emotional**	1 (0.8)	43 (35.5)	34 (28.1)	43 (35.5)	33.63 ± 11.78
**Total QOL**	2 (1.7)	63 (52.1)	48 (39.7)	8 (6.6)	94.35 ± 26.22

Mean quality of life score, SD = standard deviation, QOL = quality of life, No. (%) = number (percent)

The distributions of QOL data were not normal. Therefore, Mann-Whitney U tests were used to compare QOL scores between patients without stereoscopic vision and those with VF diameters of 20 degrees or less. The results of the Mann-Whitney U test indicated that participants without stereoscopic vision had significantly lower QOL scores in the social (P = 0.003) and leisure (P = 0.009) domains than other participants. Participants with VF diameters of 20 degrees or less scored significantly lower on the mobility and self-care QOL domains (P < 0.001) than participants with VF diameters of more than 20 degrees (Table 6).

**Table 6:** Result of Mann-Whitney U tests used to compare different QOL domains between patients with or without stereopsis or VF diameters off 20 degrees or less

	**Emotional Health**	**Social Life**	**Mobility**	**Leisure**	**Self-care**
**Stereopsis**					
Mann-Whitney U	411.5	200.0	326.5	241.0	383.5
P	0.36	0.0	0.08	0.01	0.23
**VF**					
U Mann-Whitney	1493.5	1488.5	968.5	1580.0	966.0
P	0.2	0.2	< 0.001	0.43	< 0.001

VF = visual field, QOL = quality of life

## DISCUSSION

The goal of rehabilitation programs is to assess and improve QOL in individuals with disabilities (15). QOL is influenced by various factors, such as social, economic, and cultural status, and physical health (16). Determining the impacts of various factors associated with impaired vision on the QOL of individuals with low vision and blindness in different countries and cultures is necessary and very important. The province of Sistan and Baluchestan in southwestern Iran has particular social and cultural conditions due to poverty and deprivation in the area. The current study is the first investigation of QOL factors affected by low vision and blindness in the area. 

Our results indicate that individuals without stereopsis scored lower on the social and leisure domains of QOL than normal people. Kuang et al. have reported that general health is significantly worse in individuals without stereopsis than in others and that defects in stereopsis have significant effects on the vitality dimension of the 36-Item Short Form Health Survey. Interestingly, their report indicated that defective stereopsis does not have adverse effects on visual function (13). Their results are not consistent with this study, probably because the subjects in that study were elderly individuals and were self-selected. Elderly individuals may have adapted to reduced stereopsis through experience or by using monocular and size clues. Other research by Datta et al. indicated that stereopsis is strongly associated with physical activity (17). Considering that defective stereopsis affects vision and impacts QOL, this factor should be considered by policy makers when planning healthcare strategies.

Here we observed a significant reduction in scores on the self-care and mobility domains of QOL in individuals with tunnel vision. Studies by McKean-Cowdin and Patino indicated that individuals with VF impairment experience a lower QOL and that defects affecting the VF decrease a person’s QOL (18, 19). A study by Richard on individuals with glaucoma indicated that there is a modest correlation between driving, general vision, and VF impairment. The early stages of glaucoma do not usually produce symptoms and did not have any strong correlations with VF and QOL (20). The effects of VF deficits on an individual’s QOL are unsurprising, especially those on the mobility and self-care domains. Individuals with tunnel vision have difficulty avoiding obstacles and performing visual searches (21). Kuyk et al. reported that defective VF and tunnel vision affect an individual’s mobility and orientation (22). The absence of mobility and self-care are known to lead to difficulties in social integration and social isolation. The above information regarding the effects of VF deficits highlights important factors to consider in rehabilitation services. Demographic factors, such as sex, income, and education, are known to influence QOL (23, 24). Our results indicated that QOL was significantly lower in women than in men. Similarly, Kranciukaite et al. and Yun et al. reported that women had lower QOL scores than men (25, 26). However, studies by Fernandez et al. and Nejati and Ashayeri indicated that there is no significant association between sex and QOL (27, 28).

The effects of different factors on QOL may vary in different communities. For example, some communities provide more limited opportunities for outdoor physical activity than others do. In addition, it has been suggested that women are more sensitive to adverse events than men are. There are other cultural and social factors that may contribute to the lower QOL in women (28). In Sistan and Baluchestan Province, blindness has a larger impact in women than in men due to the specific regional culture, as well as poverty and deprivation in the area. This highlights the need for sex to be a major consideration in designing rehabilitation programs. We observed significant differences in QOL scores between employed and unemployed individuals. This result is inconsistent with Amini et al.'s study, which reported that employment and QOL have no significant correlation (P = 0.241) (29). Amini et al. investigated blind war veterans who benefited from financial support from the Martyrs and Veterans Affairs Foundation. The results of this study are consistent with Lis's study, which reported a significant correlation between QOL and employment status (30). Wexler concluded that a patient’s income has a significant effect on his or her QOL. It was reported that patients with low income have many problems, such as those related to spirituality and feelings of self-worth within the family and community (31). The impacts of employment, social presence, and financial independence as social determinants of health may improve QOL in individuals. As the majority of the population in this study were unemployed and had low educational status, planning for employment and increasing the population’s level of education are essential factors in improving QOL. One limitation of our study is that because of their blindness, the patients could not self-administer the questionnaire. The questions were thus read aloud and the patients’ responses were recorded. This technique may have introduced a bias that affected the patients’ responses. Of course, the questions were read for each patient individually. 

In conclusion, based on the results of this study, we recommend the promotion of education, community participation, and leisure programs in addition to providing rehabilitation services, training for mobility, self-care, and daily activities for individuals with blindness and low vision, especially women. More research in this field, including international collaborations, would be beneficial in supporting individuals with blindness and low vision in this deprived area.
